# Data Resource Profile: Cheeloo Lifespan Electronic-health reseArch Data-library (Cheeloo LEAD)

**DOI:** 10.1093/ije/dyag091

**Published:** 2026-06-15

**Authors:** Shuaijie Zhang, Weiwei Chi, Qing Wang, Lei Li, Yongchao Wang, Qi Zhang, Hongkai Li, Xifeng Hu, Qingbo Zhao, Yuanyuan Yu, Xiaoru Sun, Lei Hou, Ping Su, Zhongshang Yuan, Xiaokang Ji, Fuzhong Xue

**Affiliations:** Department of Medical Dataology, School of Public Health, Cheeloo College of Medicine, Shandong University, Jinan, 250012, China; National Institute of Health and Medical Big Data, Jinan, 250003, China; Shandong Provincial Health Commission, Jinan, 250002, China; Department of Medical Dataology, School of Public Health, Cheeloo College of Medicine, Shandong University, Jinan, 250012, China; National Institute of Health and Medical Big Data, Jinan, 250003, China; National Administration of Health Data, Jinan, 250002, China; Department of Medical Dataology, School of Public Health, Cheeloo College of Medicine, Shandong University, Jinan, 250012, China; National Institute of Health and Medical Big Data, Jinan, 250003, China; Department of Medical Dataology, School of Public Health, Cheeloo College of Medicine, Shandong University, Jinan, 250012, China; National Institute of Health and Medical Big Data, Jinan, 250003, China; Department of Medical Dataology, School of Public Health, Cheeloo College of Medicine, Shandong University, Jinan, 250012, China; National Institute of Health and Medical Big Data, Jinan, 250003, China; Department of Biostatistics, School of Public Health, Cheeloo College of Medicine, Shandong University, Jinan, 250012, China; Department of Medical Dataology, School of Public Health, Cheeloo College of Medicine, Shandong University, Jinan, 250012, China; National Institute of Health and Medical Big Data, Jinan, 250003, China; Department of Medical Dataology, School of Public Health, Cheeloo College of Medicine, Shandong University, Jinan, 250012, China; National Institute of Health and Medical Big Data, Jinan, 250003, China; Department of Medical Dataology, School of Public Health, Cheeloo College of Medicine, Shandong University, Jinan, 250012, China; National Institute of Health and Medical Big Data, Jinan, 250003, China; Department of Medical Dataology, School of Public Health, Cheeloo College of Medicine, Shandong University, Jinan, 250012, China; National Institute of Health and Medical Big Data, Jinan, 250003, China; Department of Medical Dataology, School of Public Health, Cheeloo College of Medicine, Shandong University, Jinan, 250012, China; National Institute of Health and Medical Big Data, Jinan, 250003, China; National Administration of Health Data, Jinan, 250002, China; National Institute of Health and Medical Big Data, Jinan, 250003, China; Department of Biostatistics, School of Public Health, Cheeloo College of Medicine, Shandong University, Jinan, 250012, China; Department of Medical Dataology, School of Public Health, Cheeloo College of Medicine, Shandong University, Jinan, 250012, China; National Institute of Health and Medical Big Data, Jinan, 250003, China; Department of Medical Dataology, School of Public Health, Cheeloo College of Medicine, Shandong University, Jinan, 250012, China; National Institute of Health and Medical Big Data, Jinan, 250003, China; Qilu Hospital, Cheeloo College of Medicine, Shandong University, Jinan, 250012, China

**Keywords:** electronic health records, data resource, data linkage, lifespan epidemiology

Key Features

- Cheeloo Lifespan Electronic-health reseArch Data-library (Cheeloo LEAD) is the largest population-based electronic health record (EHR) data source in China, which serves as a longitudinal and multicenter resource to advance life-course research.
- Cheeloo LEAD was constructed through stratified cluster random sampling across Shandong Province. It comprises EHRs for 5 152 597 individuals collected from 2009 to 2024, integrating data from primary, secondary, and tertiary healthcare institutions across 39 counties and districts.
- Cheeloo LEAD provides comprehensive individual-level data, including demographic characteristics, clinical diagnoses, medication records, surgical procedures, and laboratory test results. Each participant is assigned a unique identifier, enabling linkage to external data sources such as disease-specific registries, the mortality registry maintained by the Center for Disease Control and Prevention, and regional health information platforms, including regional EHR data centers and data-sharing systems that connect healthcare institutions within the region. Furthermore, Cheeloo LEAD can be linked to environmental monitoring platforms that provide meteorological data, green-space-coverage metrics, and air-pollution indicators.
- The resource currently has a median follow-up of 13.0 years.
- Applications for access to the Cheeloo LEAD data can be submitted to xuefzh@sdu.edu.cn.

## Data resource basics

### Background

Life-course research provides essential longitudinal evidence for understanding disease trajectories and informing precision medicine [1, 2]. A fundamental requirement of this research paradigm is the availability of long-term, continuous, and large-scale data. In this context, electronic health records (EHRs) have emerged as a critical resource, enabling the systematic and cost-effective capture of individual health events across real-world clinical settings [3–6]. Several high-income countries, including the USA and the UK, have established longitudinal EHR-based cohort databases to facilitate life-course health research [7–9]. Given its large population, China likewise faces a pressing need to develop a nationally representative, large-scale EHR database to support long-term population-health studies.

Since 2009, national healthcare informatization reforms have accelerated the development of a tiered healthcare delivery system in China, supported by EHR infrastructures aimed at establishing comprehensive health records for every individual [10]. Within this system, primary care providers such as general practitioners in community health centers and township hospitals are primarily responsible for the management of common conditions [10]. This foundational tier systematically captures key early-life and routine health events, including pediatric wellness visits, immunizations, and preventive health screenings. Patients requiring more complex care are referred to secondary or tertiary hospitals, where detailed diagnostics, surgical procedures, laboratory tests, and imaging examinations further enrich the health data. The nationwide unified personal identification system, together with the standardization of EHR platforms, enables the integration of health records across all levels of care [11]. This facilitates the longitudinal tracking of health events from birth to death and provides a foundation for life-course research.

The Cheeloo Lifespan Electronic-health reseArch Data-library (Cheeloo LEAD) was established by the National Institute of Health and Medical Big Data. This institute is a key component of the National Health Medical Data North Center, which is China’s first physically operational national health big-data platform authorized by the National Health Commission.

Cheeloo LEAD benefits from being established in Shandong Province, which offers distinct advantages for health research. Shandong is a national leader in the health informatics infrastructure [12], featuring a comprehensive three-tier healthcare delivery system interconnected through a pioneering provincial health information platform [13]. This system ensures extensive service coverage and continuous data capture across all levels of care. The high degree of standardization and interoperability within its EHR systems provides a robust foundation for developing a high-quality, sustainable life-course health data resource. In addition to its informatics strengths, Shandong has a demographically stable population of >100 million residents, characterized by a low rate of outmigration, which minimizes attrition in longitudinal studies [14]. The diverse geography of the province, encompassing coastal, inland, plain, and mountainous regions, further enhances the representativeness of its population [15].

### Cheeloo LEAD

**Figure 1:**
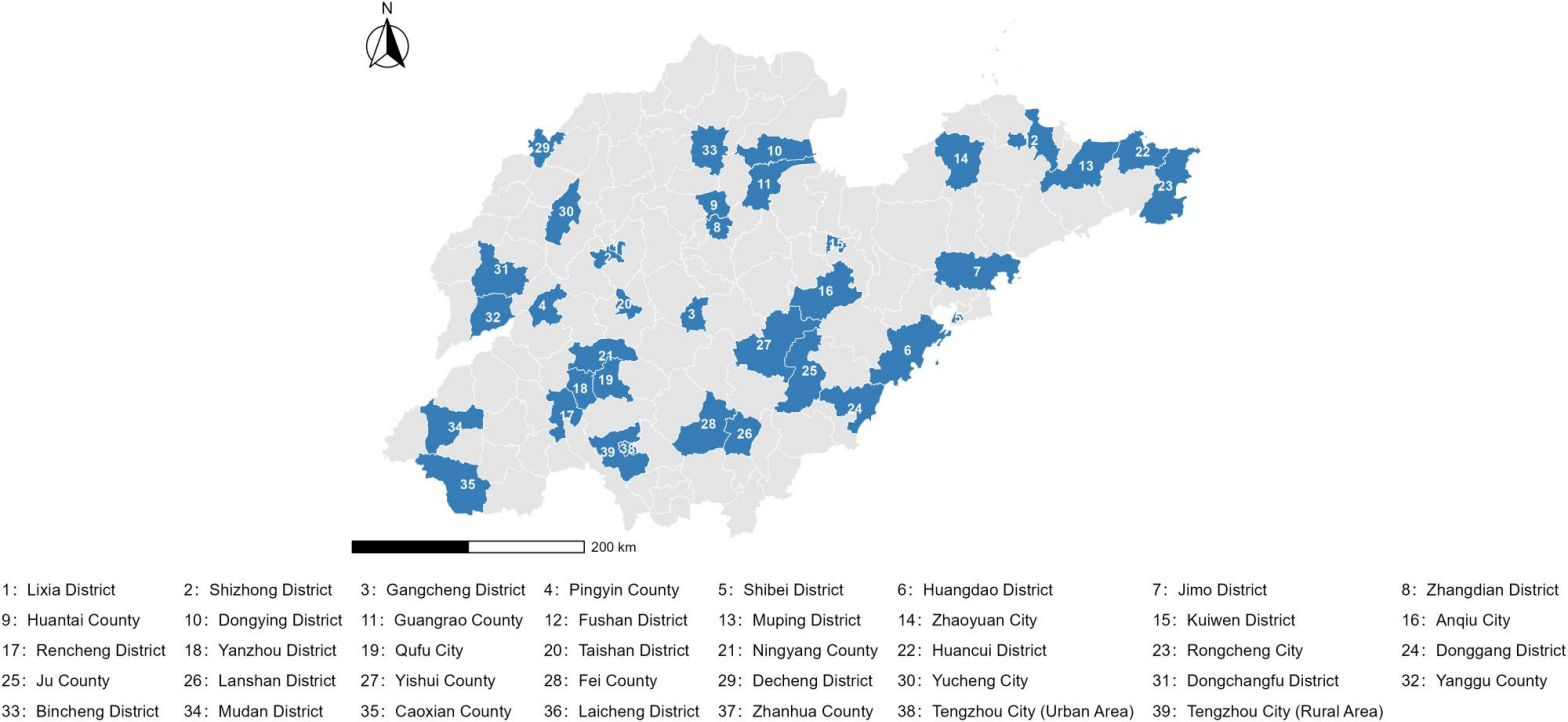
Geographical distribution of study participants in Cheeloo LEAD. The 39 sampled administrative areas across Shandong Province are highlighted.

Cheeloo LEAD is the largest anonymized EHR resource in China for life-course epidemiological research. The data originate from a province-wide consortium of healthcare institutions spanning 39 urban areas in Shandong Province (Fig. 1). It includes longitudinal health data from 5 152 597 individuals, collected between January 2009 and November 2024, with a median follow-up of 13.0 years [interquartile range (IQR): 9.9–14.3]. Importantly, the dataset enables the deterministic linkage of each participant’s health records from all contributing institutions through a unique encrypted identifier, ensuring comprehensive and continuous longitudinal records. The main demographic characteristics of the data source are summarized in Table 1.

**Table 1:** Demographic characteristics of all study participants in Cheeloo LEAD.

Characteristic	All patients
Number of patients	5 152 597
Age (years) [*n* (%)]	
<5	490 682 (9.5)
5–17	748 865 (14.5)
18–59	3 222 126 (62.5)
60+	690 924 (13.4)
Sex [*n* (%)]	
Female	2 660 138 (51.6)
Male	2 492 459 (48.4)
Region [*n* (%)]	
Rural	2 384 688 (46.3)
Urban	2 767 909 (53.7)
Follow-up in years [median (IQR)]	13.0 (9.9–14.3)

## Data collected

### Data extraction

To ensure the representativeness of the study population, a stratified multistage cluster sampling strategy was employed. Sampling was stratified across five predefined subcohorts: rural community residents, urban community residents, urban occupational groups, students, and mothers and young children. Participants were included from 39 randomly selected administrative areas. Participant inclusion relied on health information systems, including resident health-record systems for rural and urban community residents; hospital information systems of tertiary hospitals for urban occupational groups, restricted to individuals with at least three health examinations at the same institution; the Student Health Examination Database for students; and the Maternal and Child Health Management System for mothers and young children. Detailed sampling procedures are described in the Supplementary Materials.

**Figure 2:**
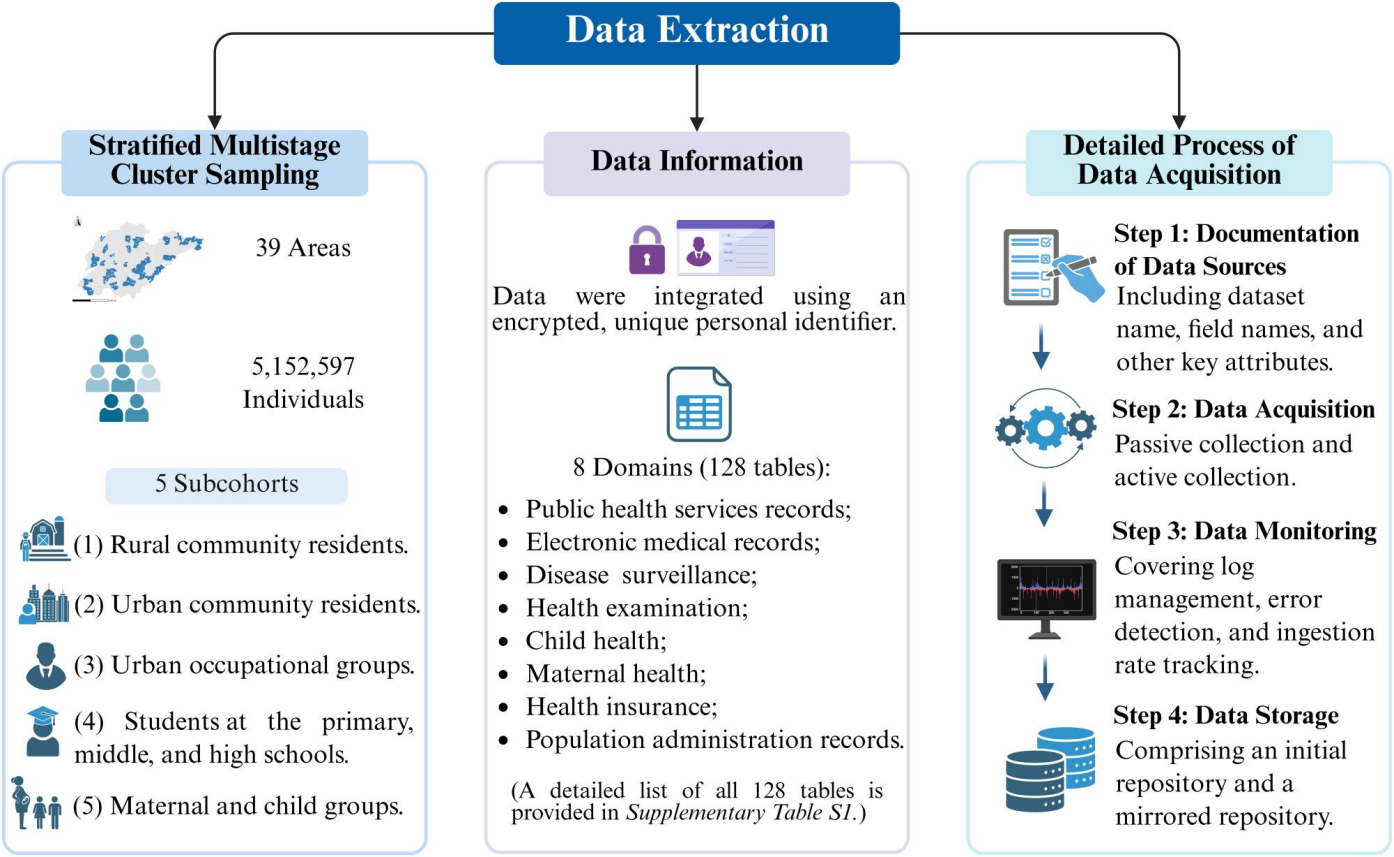
The framework of data extraction [created in BioRender, Zhang, S. (2026), https://BioRender.com/u980sbu].

Data acquisition was performed on a secure government extranet, adhering to stringent security and data-protection protocols. The process involved four key stages (Fig. 2). First, pre-acquisition protocols included the systematic registration of all data sources, documentation of detailed metadata for each table (e.g. field names and attributes), and routine testing of database connectivity to ensure stability. Second, data were collected by using two complementary approaches. Passive data collection was implemented for secondary and tertiary healthcare institutions, in which mature EHR integration platforms enable source systems to push updates to the central EHR platform in near real time through preconfigured interfaces. Active data collection was conducted via scheduled extract–transform–load procedures [16] to supplement information that could not be captured through passive collection—particularly data from primary care facilities, death registries, and public health systems. Active collection is performed annually and each cycle requires a formal data-extraction application followed by approval from the relevant governmental data-management authorities. Third, continuous quality control (QC) was maintained by monitoring scripts that tracked data flows, managed logs, and detected errors. Finally, data were stored in two distinct repositories. An initial repository held raw and unaltered source data to ensure full traceability. The second repository served as a mirrored version, consolidating these data into a unified platform while preserving the integrity of the original records during subsequent data-governance processes.

### Data governance

**Figure 3:**
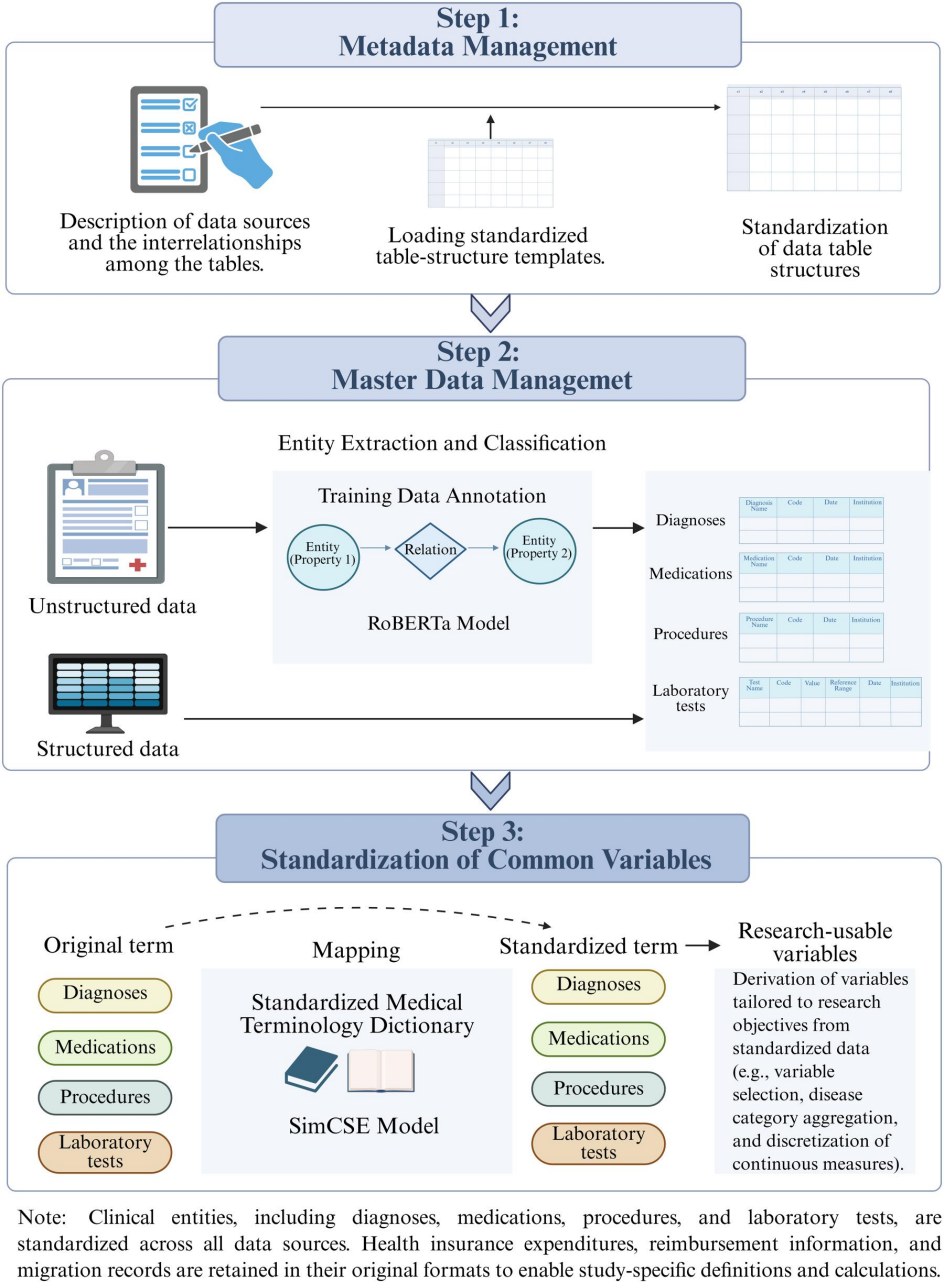
The data-governance workflow [created in BioRender, Zhang, S. (2026) https://BioRender.com/22ofm23].

The data-governance framework was based on the Observational Medical Outcomes Partnership (OMOP) Common Data Model (CDM) [17]. Its implementation involved three key steps, as shown in Fig. 3: metadata management, master data management, and the standardization of common variables.

#### Step 1: Metadata management

Metadata management was designed to ensure clear data provenance and to enforce standardized table structures from heterogeneous sources. The process begins with the detailed documentation of each data table, covering its source, content type, and associations with other tables. Cross-table linkages are then established by using a variety of fields, such as unique patient identifiers, admission IDs, and timestamps. Subsequently, standardized table templates developed in accordance with national and provincial specifications were loaded into the platform to standardize the data structure. These templates specify the required data elements, formats, and data types for core datasets, such as inpatient and outpatient records, medical-record front pages, and laboratory reports. Finally, data harmonization transforms tables from heterogeneous sources to align with standard templates, eliminating structural discrepancies and enabling data interoperability for analysis.

#### Step 2: Master data management

Master data management integrated heterogeneous datasets from both structured data and unstructured clinical text into four domains: diagnoses, medications, procedures, and laboratory tests. Structured data were extracted directly from the corresponding domain tables. Unstructured text was processed by using the RoBERTa model [18] to recognize and extract the relevant entities. All four datasets contain common fields, including encrypted patient identifiers and hospital names, while each also features specific key information: disease data contained diagnoses name, diagnoses code, and diagnoses date; medication data included medication name, medication code, and prescription date; procedure data contained procedure name, procedure code, and procedure date; and laboratory test data included test name, test code, test result, reference range, and test date.

#### Step 3: Standardization of common variables

To ensure semantic interoperability, the supervised SimCSE model [19] was utilized to standardize the nomenclature and coding of all extracted entities by mapping them to reference dictionaries. This process yielded 31262 distinct disease and related health variables (ICD-10), 5836 medication variables, 7305 procedure variables, and 752 laboratory test variables.

### Data quality

A rigorous multi-level quality-control (QC) framework was applied across the data processing to ensure accuracy and consistency. It comprised three stages: acquisition QC, structural and relational QC, and semantic QC. During acquisition QC, unannounced on-site audits in randomly selected regions were conducted to assess the quality and authenticity of the health-record data. The completeness of all required tables and fields were also verified, data types and formats were validated, and collected records were reconciled with the original source documents to ensure consistency. Following metadata mapping, structural and relational QC assessed conformance with standard table structures. Checks included quantifying missingness for key variables such as age and sex, evaluating cross-field consistency and overall data integrity, and confirming correct linkage via the encrypted patient identifier. Semantic QC then evaluated the accuracy of the RoBERTa and SimCSE models used for data standardization. Automated coding was accepted at a similarity threshold of 0.80. Mappings below this threshold underwent manual review. Categories with high error rates received additional manual verification and secondary cross-checking to ensure coding precision.

### Data linkages

Shandong Province has established a unified and interoperable provincial health information platform. Data linkage for Cheeloo LEAD can be conducted through the provincial e-government platform, operating within a strict framework of ethical approval and data security. Individual-level records from multiple healthcare institutions are deterministically linked through a unique encrypted personal identifier. Cheeloo LEAD leverages this identifier to integrate 128 distinct tables (Supplementary Table 1) categorized into eight domains: public health services records and electronic medical records comprise community-based health archives and hospital clinical encounter data, respectively. Disease surveillance encompasses registries for notifiable infectious diseases and chronic-condition management. The remaining domains include health examination, maternal and child health, health insurance, and population administrative data. The specific measures are summarized in Table 2. Additionally, Cheeloo LEAD can be linked to regional health information platforms and environmental monitoring systems covering meteorology, green space, and air pollution, thereby facilitating comprehensive research.

**Table 2:** Summary of linked data domains and key information in Cheeloo LEAD.

Data domain	Key information and measures
Public health services records	Demographics (age, sex, ethnicity, education, occupation, marital status), blood type, and medical history (past diagnoses, surgeries, transfusions, family history)
Electronic medical records	Clinical encounters (inpatient, outpatient, emergency visits, referrals), diagnoses and interventions (disease codes, procedures, prescriptions), laboratory results, and imaging reports
Disease surveillance	Chronic-disease management (hypertension, diabetes, stroke, coronary heart disease, severe mental disorders, including medication, symptoms), infectious-disease surveillance (tuberculosis, HIV/AIDS, hepatitis B, notifiable-disease reports), cancer registries, vital statistics (medical certificate of death, cause of death), and public health monitoring (vaccination records, occupational disease)
Health examination	General physical examination (anthropometrics, blood pressure, vision, hearing, lifestyle factors), student health examination, and occupational health examination results
Child health	Birth-registration details (birthweight, gestational age, maternal information), neonatal-disease screening results, birth-defects surveillance (defect type, diagnostic basis), neonatal follow-ups (weight change, danger signs, referral flags), child health examinations (somatic growth assessment, height, weight, vision, hearing, lung and heart findings), and neonatal-death records
Maternal health	Delivery information (mode of delivery, gestational age, pregnancy outcome) and reproductive-health history (parity, gynecologic screening for cervix and breast)
Health insurance	Insurance enrollment (insurance type, locality) and medical claims (total cost, out-of-pocket expenses, settlement time, itemized costs, and payment categories)
Population administration records	Vital registration and migration (marriage registration, birth approvals, in-migration and outmigration reasons and dates) and social welfare records (disability type and severity, incentive, and special-assistance beneficiaries)

### Data privacy and security

The management of the Cheeloo LEAD database adheres strictly to all applicable legal, regulatory, and industry standards. Each patient identifier is anonymized through a unique encrypted ID to safeguard privacy. All investigators must sign a data-confidentiality agreement before any access is granted. Data are hosted in an environment fronted by a bastion host [20] that provides a single point of entry with centralized access control. Database servers accept connections only from the Internet Protocol (IP) address of the bastion host. User authentication is required for all resources. Database administrators provision accounts in accordance with project-approval documents and enforce least-privilege access [21], granting only the permissions necessary for the approved study.

## Data resource use

Cheeloo LEAD supports real-world research across clinical and public health sciences, spanning diverse domains such as disease prediction, health economics and policy research, environmental epidemiology, prognostic assessment, and risk-factor identification.

In the domain of disease prediction, for instance, interpretable machine-learning models were built to provide cost-efficient digital prescreening for lung cancer and mental disorders [22, 23].

In health economics and policy research, the database has been used to evaluate the national breast cancer screening program, specifically highlighting its impact on narrowing the rural–urban survival gap among women aged 35–64 years [24]. Additionally, it has supported comparative analyses of inpatient care accessibility and the economic burden of lung cancer under a tiered social health insurance framework [25].

In environmental health, Cheeloo LEAD has been used to quantify rural–urban disparities in cardiovascular disease risk and the economic burden attributable to nitrogen dioxide exposure [26]. It has also supported evaluations of the effects of long-term particulate-matter exposure on type 2 diabetes incidence, complication progression, and mortality [27].

Regarding disease progression and prognosis, longitudinal data from Cheeloo LEAD have revealed associations between baseline and longitudinal trajectories of the triglyceride–glucose index and incident cardiovascular disease in older adults [28]. Furthermore, data spanning 2013–23 underpinned the construction of a dynamic model that charts the full natural history of type 2 diabetes, from health state to onset, complications, and mortality [29].

Finally, in risk-factor studies, Cheeloo LEAD has identified key determinants of acute ischaemic stroke in adults aged 18–50 years [30] and assessed the effects and interactions of multiple chronic conditions on age-related macular-degeneration risk [31].

## Strengths and weaknesses

### Strengths

Cheeloo LEAD is the largest EHR research database in China, comprising medical records for >5 million individuals obtained through cluster random sampling. Its scale enables estimations of disease incidence and mortality. It also provides exceptional statistical power for epidemiological analyses and permits the detection of modest associations that smaller cohorts may miss. These capabilities are particularly valuable for studying rare diseases.

Moreover, the database offers long-term follow-up, currently with standardized health records covering the period from 2009 to 2024. Supported by Shandong Province’s advanced health informatics infrastructure, follow-up information from secondary and tertiary hospitals can be captured as early as *T* + 1 day post-visit, enabling near real-time data acquisition. Records acquired after 2024 are currently undergoing centralized standardization and will be made available for research use once this process is complete. This longitudinal continuity supports the detailed characterization of disease trajectories and the evaluation of long-term intervention effects, particularly in health economics and policy research.

A further strength is rigorous standardization. Guided by the unified data-collection and reporting policy of the Shandong Provincial Health Commission, Cheeloo LEAD implements a consistent acquisition framework and systematically integrates diagnoses, medications, surgical procedures, and laboratory tests. Such standardization ensures cross-institutional consistency and comparability, enabling reproducible multicenter research.

Finally, the reliability of the data is enhanced through the real-time entry of clinical variables by healthcare professionals during routine care, which minimizes recall bias and strengthens the validity of research findings.

### Weaknesses

Several limitations should be noted. At present, the data originate solely from Shandong Province, which constrains national representativeness. Broader geographic coverage could be achieved through cross-regional collaborations that integrate health databases from multiple provinces. In addition, although the clinical records are rich, key health-related factors such as lifestyle, dietary habits, and family structure are not adequately recorded, as no study-specific, purpose-built questionnaires have been implemented. This limitation restricts investigations that require granular behavioral and social information. Looking ahead, unified anonymization protocols could enable secure linkage of Cheeloo LEAD’s unique identifiers with external datasets, thereby supporting broader population-health analyses.

## Data resource access

To request access to Cheeloo LEAD, investigators must submit a formal application specifying study objectives, the required data scope, period of use, and an analysis plan. All requests are reviewed by the Data Security Management Committee and applicants must execute a data-sharing and data-security agreement. Approval depends on adherence to relevant data-protection laws and on the operational feasibility of the proposal.

Upon approval, a data manager works with the investigator to translate the research question into a specification of datasets. Approved data are provisioned within a secure virtual research environment. Each investigator receives an individual account with access via a designated virtual private network (VPN). Cheeloo LEAD data scientists can provide technical support for data cleaning when required. Collaboration or research proposals should be directed to the corresponding author (xuefzh@sdu.edu.cn).

## Ethics approval

Cheeloo LEAD was approved by the Institutional Review Board of the School of Public Health, Shandong University, China. In accordance with the national standard “Information Security Technology—Personal Information Security Specification,” scientific research conducted in the public interest does not require individual consent once personal information has been de-identified.

## Supplementary Material

dyag091_Supplementary_Data

## Data Availability

The data underlying this article cannot be shared publicly due to ethical, privacy, and data-security restrictions related to individual-level electronic health records. The data will be shared on reasonable request to the corresponding author.
