# Supplementary material for: Data Resource Profile: Cheeloo Lifespan Electronic-health reseArch Data-library (Cheeloo LEAD)
Source: Int J Epidemiol. 2026 Jun 15;55(3):dyag091. doi: 10.1093/ije/dyag091 (PMC13265376; doi:10.1093/ije/dyag091)
Supplement: dyag091_Supplementary_Data [file dyag091_supplementary_data.zip › Supplementary_Materials (2).pdf]

Supplementary Materials

Selection of Administrative Areas

Shandong Province comprised 17 prefecture-level cities at the time of study design (reduced to 16 following the administrative merger of Laiwu into Jinan in December 2019). Using simple random sampling, both rural and urban administrative units were selected within each prefecture-level city. This process resulted in a total of 39 administrative areas, which together constituted the geographic sampling frame for Cheeloo LEAD (Table A1).

Table A1. Administrative areas sampled for Cheeloo LEAD

Prefecture-level cities	Rural counties	Urban districts
1. Heze	Caoxian	Mudan
2. Liaocheng	Yanggu	Dongchangfu
3. Dezhou	Yucheng	Decheng
4. Jining	Qufu, Yanzhou	Rencheng
5. Zaozhuang	Tengzhou (rural area)	Tengzhou (urban area)
6. Taian	Ningyang	Taishan
7. Laiwu	Laicheng	Gangcheng
8. Jinan	Pingyin	Lixia, Shizhong
9. Linyi	Yishui, Fei County	Lanshan
10. Zibo	Huantai	Zhangdian
11. Weifang	Anqiu	Kuiwen
12. Bingzhou	Zhanhua	Bincheng
13. Dongying	Guangrao	Dongying
14. Rizhao	Ju County	Donggang
15. Qingdao	Jimo, Huangdao	Shibei
16. Yantai	Zhaoyuan, Muping	Fushan
17. Weihai	Rongcheng	Huancui

Inclusion of Participants

Cheeloo LEAD employs the stratified multistage cluster sampling strategy to ensure the representativeness of the study population. Sampling was stratified across five predefined subcohorts: rural community residents, urban community residents, urban occupational groups, students, and mothers and young children. Participants were not recruited through individual invitation; instead, they were included through health information systems, including resident health record systems for rural and urban community residents; hospital information systems of tertiary hospitals for urban occupational groups; the Student Health Examination Database for students; and the Maternal and Child Health Management System for mothers and young children. The detailed inclusion procedures for each population group are described below.

(a) Rural community residents. Within the selected rural counties, 3–5 townships were randomly sampled. Within each sampled township, administrative villages (or residents' committees) with a permanent population of more than 1,000 were selected

using cluster sampling. All permanent residents in the selected villages were identified through the resident health record system and included as a whole. Individual identity was based on the national personal identification number, which was encrypted to generate a study-specific unique identifier for data linkage and analysis.

(b) Urban community residents. Participants were drawn from the municipal districts of the 17 prefecture-level cities. In each city, 1–2 districts were randomly selected, followed by the random selection of 5–10 communities (subdistricts) within each selected district. All permanent residents in the selected communities were identified through the resident health record system and included using the same encrypted unique identifier framework as in rural communities.

(c) Urban occupational groups. Urban occupational groups were identified from health examination centers of selected tertiary hospitals in the 17 cities. In each city, 1–3 tertiary hospitals were randomly selected. Within each selected hospital, all eligible long-term health examination participants were included. Long-term participants were defined as individuals who had completed at least three health examinations at the same center. Participants were identified through the hospital information system (HIS) and linked using encrypted personal identification numbers.

(d) Students. Students covered all 39 selected administrative areas. Within each area, all primary, middle, and high schools served as the sampling frame. Stratified cluster sampling was conducted by educational level, and entire classes were included once selected. Students were identified through the Shandong Student Health Examination Database, and encrypted personal identification numbers were used for linkage with other data sources.

(e) Mothers and young children. Participants also covered all 39 administrative areas. Mothers and young children (0–6 years old) were identified through the Maternal and Child Health Management System. All eligible individuals were included as a whole, with encrypted personal identification numbers used to support data integration and longitudinal follow-up.

To ensure data reliability, rigorous on-site quality verification was conducted under the coordination of the Shandong Provincial Health Commission. Field investigators evaluated data entry quality and completeness through interviews with data managers, while data engineers monitored real-time uploads to the central platform to identify logical errors or missing values. To verify authenticity, unannounced on-site audits were performed. Specifically, in primary care facilities, a 10% random subsample of participants underwent door-to-door verification. In addition, the observed prevalence of major chronic diseases (e.g., hypertension and diabetes) and corresponding risk factor exposure rates were compared with national epidemiological benchmarks to evaluate data plausibility. Sampling units with data distributions that substantially deviated from expected ranges and could not be explained through on-site verification were deemed to have insufficient data quality and were replaced by geographically proximate units that met quality criteria.

In accordance with the national standard *Information Security Technology—Personal Information Security Specification*, scientific research conducted in the public interest does not require individual consent once personal information has been de-identified. All data use and linkage procedures were conducted under ethical approval, legal compliance, and strict de-identification requirements.
